# Supplementary figures and images for: Follow-up of antibody changes in brucellosis patients in Gansu, China
Source: Microbiol Spectr. 2025 Apr 30;13(6):e02862-24. doi: 10.1128/spectrum.02862-24 (PMC12131799; doi:10.1128/spectrum.02862-24)

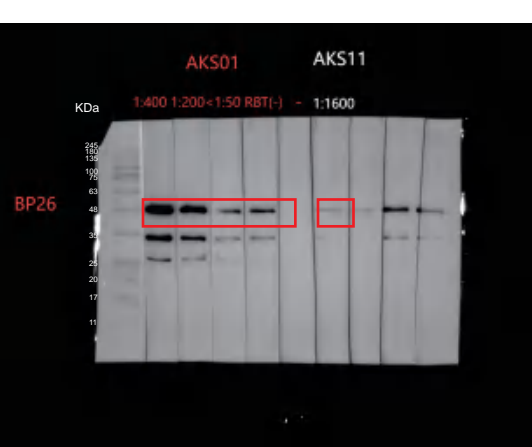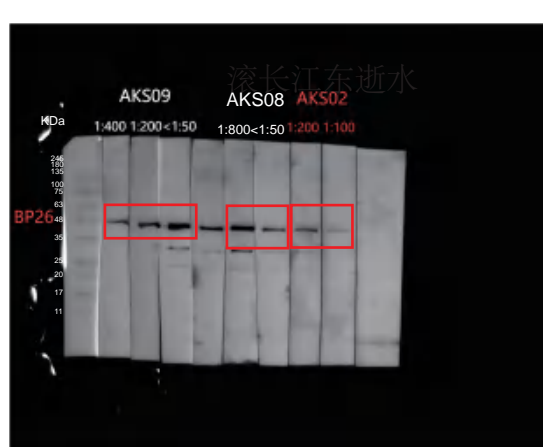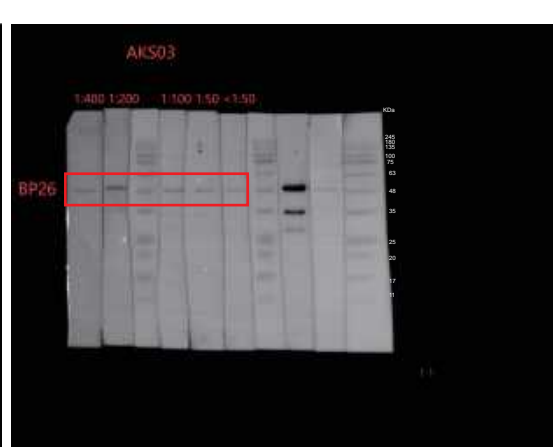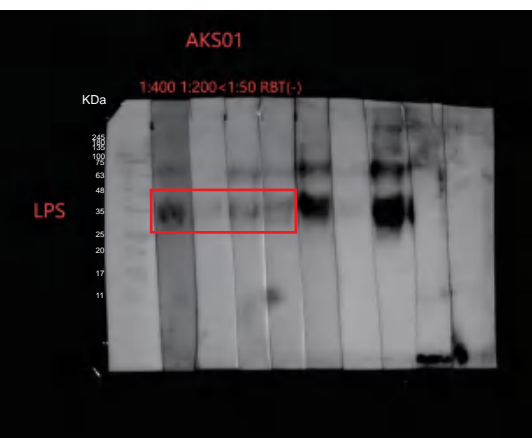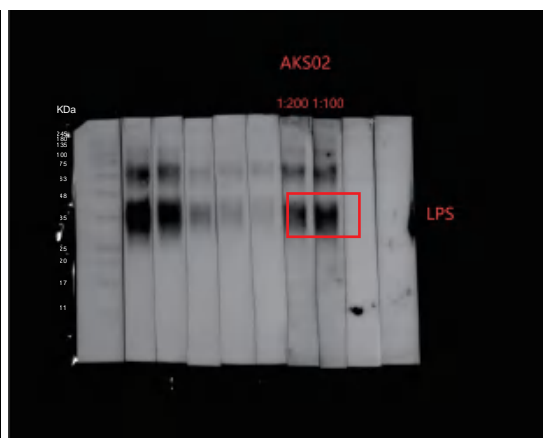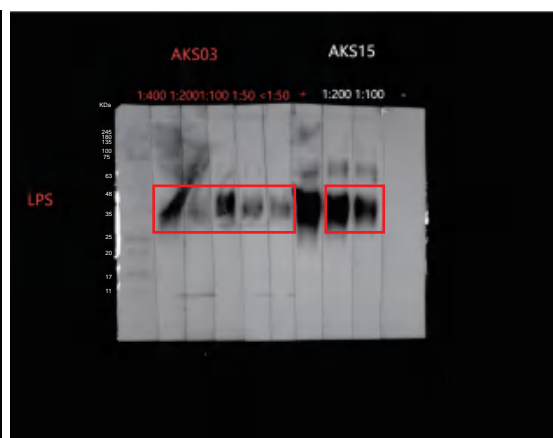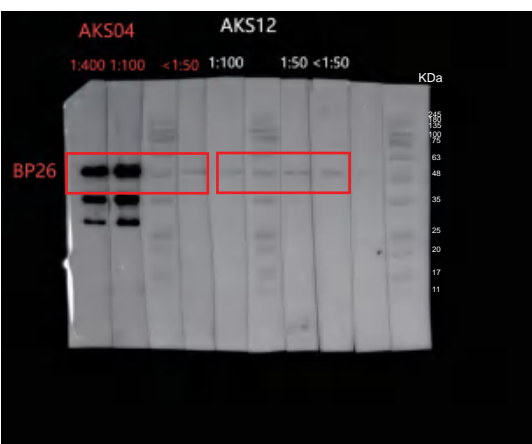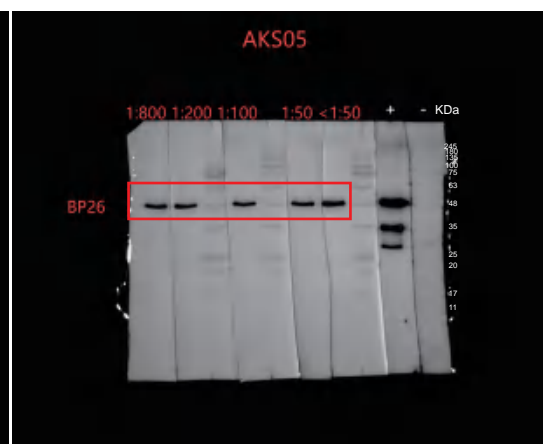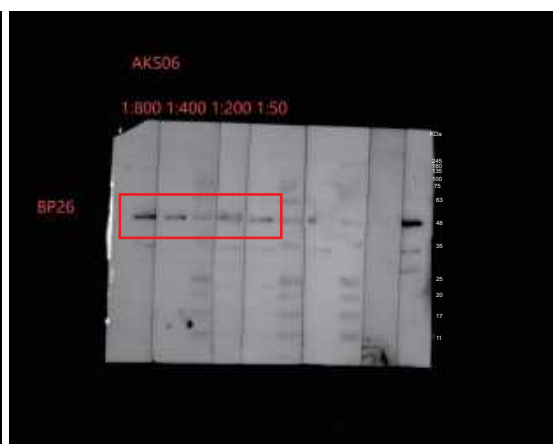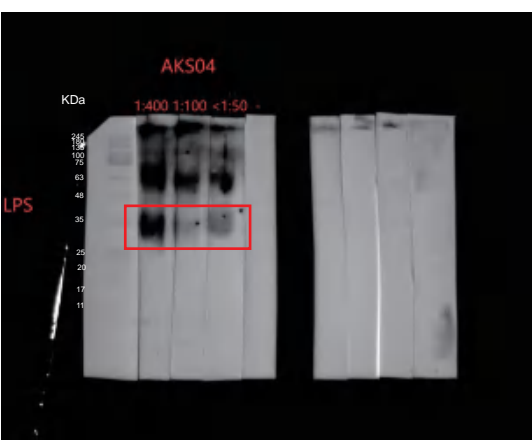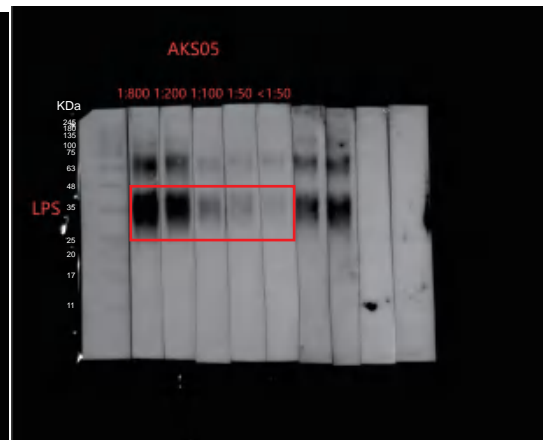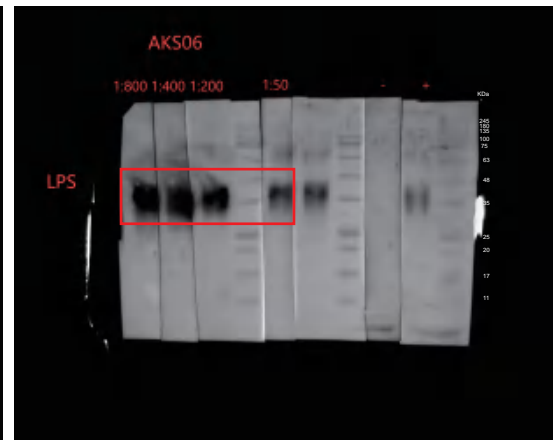

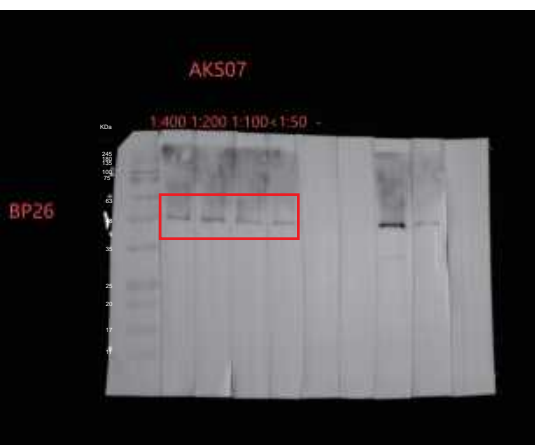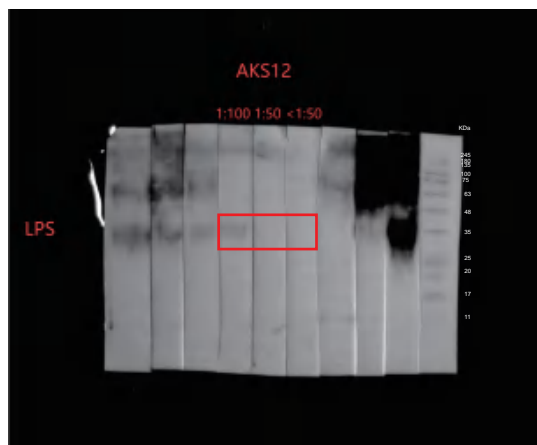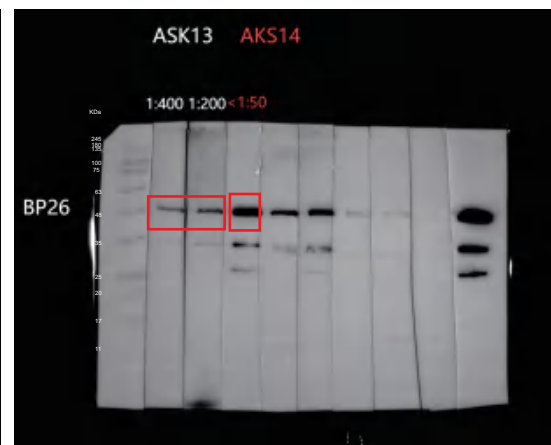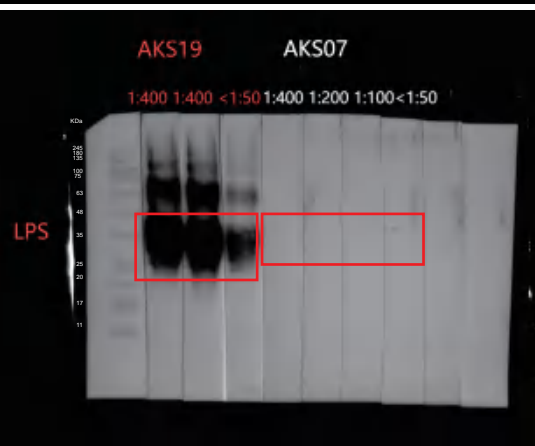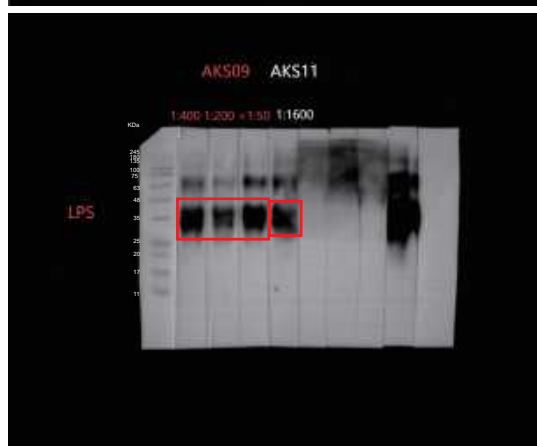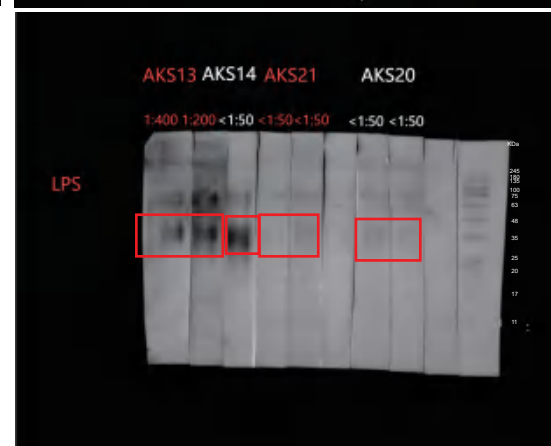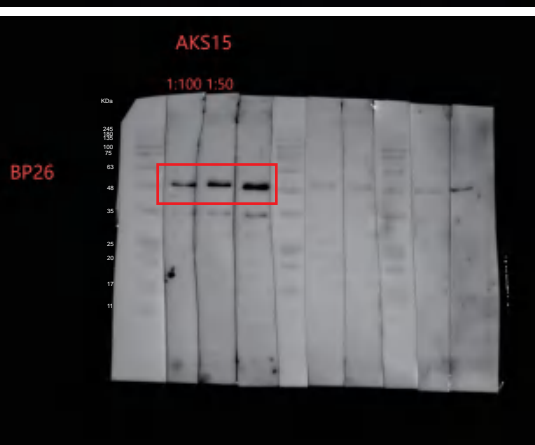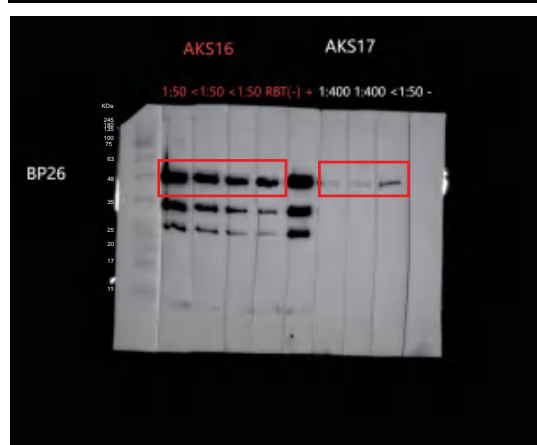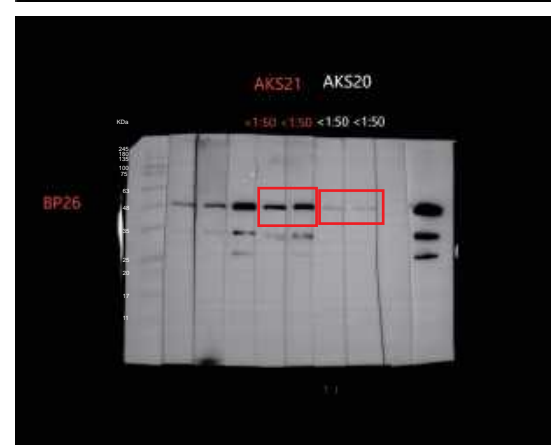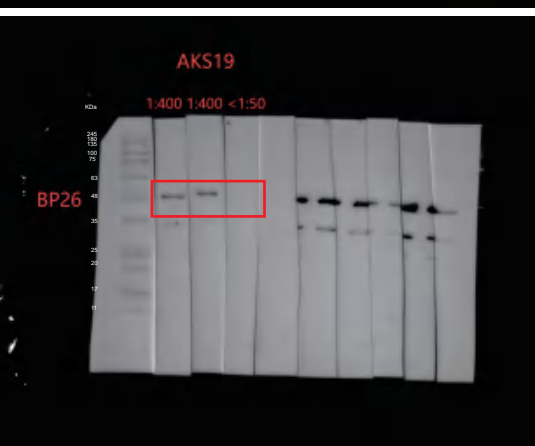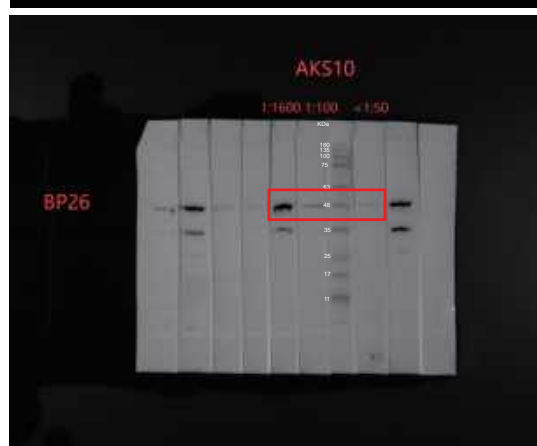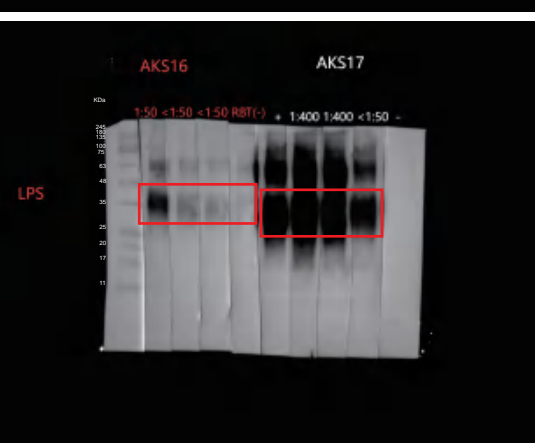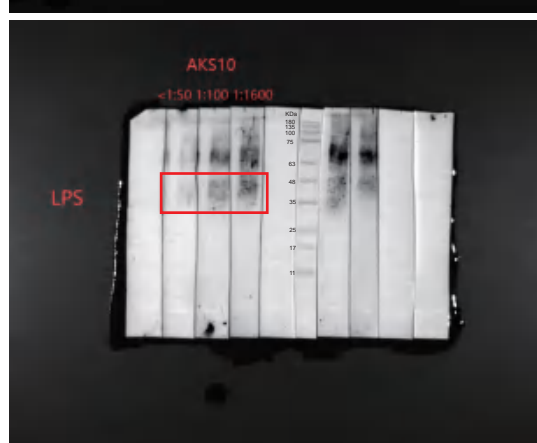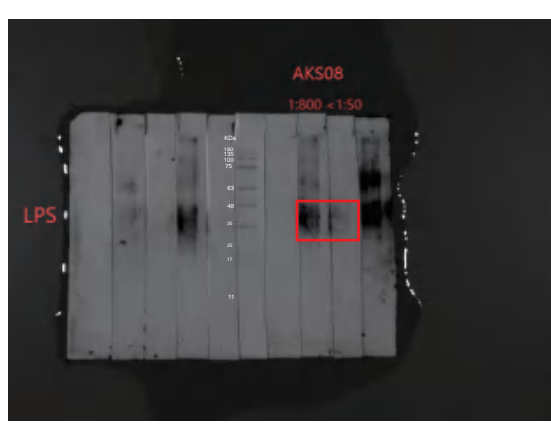

Supplement: Figure S1 — Western blot full-length image of Figure 4. [file spectrum.02862-24-s0001.pdf]
