# Supplementary material for: Follow-up of antibody changes in brucellosis patients in Gansu, China
Source: Microbiol Spectr. 2025 Apr 30;13(6):e02862-24. doi: 10.1128/spectrum.02862-24 (PMC12131799; doi:10.1128/spectrum.02862-24)
Supplement: Supplemental material legends — Legends for Figure S1 and Table S1. [file spectrum.02862-24-s0002.docx]

**Supplemental Material Legends**

**Supplementary Table S1.** Demographics table of brucellosis cases in Akesai Kazakh Autonomous County(- indicate not available，* indicate time interval between onset of symptoms and first blood collection.)

**Supplementary Figure S1.** Western blot full-length image of Figure 4
