# Supplementary material for: Follow-up of antibody changes in brucellosis patients in Gansu, China
Source: Microbiol Spectr. 2025 Apr 30;13(6):e02862-24. doi: 10.1128/spectrum.02862-24 (PMC12131799; doi:10.1128/spectrum.02862-24)
Supplement: Table S1 — Demographics table of brucellosis cases in Akesai Kazakh Autonomous County. [file spectrum.02862-24-s0003.docx]

**Supplementary Table S1.** Demographics table of brucellosis cases in Akesai Kazakh Autonomous County

| ID | Age | Gender | Occupation | Symptoms | Fever(℃) | Hyperhidrosis | Fatigue | Muscle soreness | Arthralgia | Eruption | Time interval*  (Days) | Treatment | Direct animal exposure |
| --- | --- | --- | --- | --- | --- | --- | --- | --- | --- | --- | --- | --- | --- |
| AKS01 | 62 | Male | Farmer | Yes | 38.5 | Yes | Yes | Yes | Yes | No | 49 | Yes | Yes |
| AKS02 | 34 | Male | Herdsman | Yes | 38 | No | Yes | No | No | No | 6 | Yes | Yes |
| AKS03 | 57 | Female | Farmer | Yes | 38.5 | Yes | Yes | Yes | Yes | No | 7 | Yes | Yes |
| AKS04 | 44 | Female | Farmer | Yes | Unknown | No | Yes | No | No | No | ≈21 | Yes | Yes |
| AKS05 | 42 | Male | Farmer | Yes | 38.1-39.8 | No | Yes | Yes | No | No | 10 | Yes | Yes |
| AKS06 | 43 | Male | Herdsman | Yes | Unknown | Yes | Yes | Yes | Yes | No | ≈15 | Yes | Yes |
| AKS07 | 52 | Male | Farmer | Yes | 38.8 | Yes | Yes | Yes | Yes | No | ≈14 | Yes | Yes |
| AKS08 | 38 | Male | Farmer | Yes | Unknown | No | Yes | Yes | No | No | ≈25 | Yes | Yes |
| AKS09 | 71 | Male | Herdsman | Yes | Unknown | Yes | Yes | No | No | No | ≈15 | Yes | Yes |
| AKS10 | 36 | Male | Farmer | Yes | Unknown | No | Yes | No | No | No | ≈25 | Yes | Yes |
| AKS11 | 59 | Male | Herdsman | Yes | 38.5 | No | No | Yes | Yes | No | ≈25 | Yes | Yes |
| AKS12 | 52 | Male | Veterinarian | Yes | 38.5 | Yes | Yes | No | Yes | No | 12 | Yes | Yes |
| AKS13 | 46 | Male | Farmer | Yes | Unknown | Yes | Yes | No | No | No | ≈20 | Yes | Yes |
| AKS14 | 49 | Male | Farmer | No | No | No | No | No | No | No | - | Yes | Yes |
| AKS15 | 22 | Male | Farmer | No | No | No | No | No | No | No | - | Yes | Yes |
| AKS16 | 61 | Male | Farmer | No | No | No | No | No | No | No | - | Yes | Yes |
| AKS17 | 56 | Male | Herdsman | Yes | 38 | No | Yes | Yes | No | No | ≈15 | Yes | Yes |
| AKS18 | 27 | Male | Farmer | No | No | No | No | No | No | No | - | Yes | Yes |
| AKS19 | 49 | Male | Herdsman | Yes | 38 | No | Yes | Yes | No | No | ≈15 | Yes | Yes |
| AKS20 | 48 | Male | Farmer | No | No | No | No | No | No | No | - | Yes | Yes |
| AKS21 | 44 | Male | Farmer | No | No | No | No | No | No | No | - | Yes | Yes |

- indicate not available，* indicate time interval between onset of symptoms and first blood collection.
